# Inonotus obliquus attenuates histamine-induced microvascular inflammation

**DOI:** 10.1371/journal.pone.0220776

**Published:** 2019-08-22

**Authors:** Sumreen Javed, Kevin Mitchell, Danielle Sidsworth, Stephanie L. Sellers, Jennifer Reutens-Hernandez, Hugues B. Massicotte, Keith N. Egger, Chow H. Lee, Geoffrey W. Payne

**Affiliations:** 1 Biochemistry and Molecular Biology Program, University of Northern British Columbia, Prince George, Canada; 2 Northern Medical Program, University of Northern British Columbia, Prince George, Canada; 3 Centre for Heart Lung Innovation & Department of Radiology, University of British Columba & St. Paul’s Hospital, Vancouver, Canada; 4 Ecosystem Science and Management Program, University of Northern British Columbia, Prince George, Canada; University of PECS Medical School, HUNGARY

## Abstract

Cell-to-cell communication is a key element of microvascular blood flow control, including rapidly carrying signals through the vascular endothelium in response to local stimuli. This cell-to-cell communication is negatively impacted during inflammation through the disruption of junctional integrity. Such disruption is associated with promoting the onset of cardiovascular diseases as a result of altered microvascular blood flow regulation. Therefore, understanding the mechanisms how inflammation drives microvascular dysfunction and compounds that mitigate such inflammation and dysfunction are of great interest for development. As such we aimed to investigate extracts of mushrooms as potential novel compounds. Using intravital microscopy, the medicinal mushroom, *Inonotus obliquus* was observed, to attenuate histamine-induced inflammation conducted vasodilation in second-order arterioles in the gluteus maximus muscle of C57BL/6 mice. Mast cell activation by C48/80 similarly disrupted endothelial junctions and conducted vasodilation but only histamine was blocked by the histamine antagonist, pyrilamine not C48/80 suggesting the importance of mast cell activation. Data presented here supports that histamine induced inflammation is a major disruptor of junctional integrity, and highlights the important anti-inflammatory properties of *Inonotus obliquus* focusing future assessment of mast cells as putative target for *Inonotus obliquus*.

## Introduction

Inflammation is a protective mechanism that is activated to combat invading pathogens or to reverse tissue injury. The activation of inflammatory pathways stimulates the release of pro-inflammatory mediators including nitric oxide, reactive oxygen species, interleukins (eg. IL-1, I-L6), tumor necrosis factor-alpha (TNF-α), cyclo-oxygenase (COX-2), prostaglandins (PGE2), nuclear factor (NF-κβ)[1]. Although the initial inflammatory response is protective, a chronic and unresolved response can result in cellular damage and facilitates the pathophysiology of various inflammatory mediated metabolic and neurodegenerative diseases. Therefore, mechanisms to attenuate aberrant inflammatory responses would be beneficial mitigating the onset of disease states.

The use of natural products in treating many human diseases has been extensively explored [2], with fungi, specifically mushrooms, which are the fruiting bodies of fungi, being a primary source of in recent drug development [3]. Previous studies have demonstrated the role of mushrooms in reducing inflammation in both *in-vitro* and *in-vivo* conditions [4]. Some of the common mushroom species well known for anti-inflammatory effects are *Cordyceps sinensis* (Berk.) Sacc., *Ganoderma lucidum* (Curtis) P. Karst, *Agaricus blazei* Murrill, *Cyathus africanus* H.J. Brodie and *Inonotus obliquus* (Ach. ex Pers.) Pilát [4–6]. *Inonotus obliquus*, commonly known as Chaga, has been previously shown to have anti-inflammatory activity in both *in vitro* and *in vivo* models [7–11].

One of the most potent inflammatory agents is histamine and released from mast cells in response to tissue injury or other inflammatory conditions [12,13]. Within the vasculature, histamine has been shown to be a permeability factor in the microvasculature, acting specifically on the H_1_ receptor in both endothelium and smooth muscle [12]. However, beyond histamine, few inflammatory mediators have been well characterized for their effect on conducted vasodilation. Additionally, little is known regarding arteriolar response to other specific or global inflammatory mediators. Mast cells are key regulators of inflammation, capable of releasing pre-packaged inflammatory mediators from intracellular stores. Further, the location of mast cells at the host-environment interface provides mast cells a crucial role in effective immune response during infection including the release of inflammatory mediators such as histamine [12]. Mast cells have a prominent effect on the microvasculature, as several products of degranulation serve to increase vascular permeability and regional edema [14–16]. One compound of interest as a is compound 48/80 (C48/80). As a mast cell degranulator, characterization of C48/80 will provide evidence as to whether inflammatory deterioration of conducted vasodilation is specific to histamine or if it is a global inflammatory phenomenon. Many areas of research have uncovered a relationship between inflammation and disease states. Multiple sclerosis, inflammatory arthritis, sepsis, atherosclerosis, obesity and diabetes have all shown some degree of correlation with mast cell activity [17–22]. As such, the characterization of mast cells may uncover a means by which prolonged mast cell-orchestrated immune responses may impede normal tissue function. Previous work has demonstrated a linked between mast cell activation and medicinal mushrooms such as *I*. *obliquus* [23].

Despite these findings, much remains unknown about the anti-inflammatory potential of *I*. *obliquus* or potential signaling targets. Therefore, in the present study, we sought to further investigate impact of *I*. *obliquus* on key aspects of inflammatory response using a murine model; specifically, we hypothesize that *I*. *obliquus* extracts would actively reduce mast-cell activation, and thereby inflammation, associated vascular dysfunction and whether *I*. *obliquus* reverses and mitigates microvascular inflammation in response to histamine. Assessing impact and potential signaling targets of *I*. *obliquus* will be instrumental in development of new anti-inflammatory therapeutics from medicinal mushrooms.

## Materials & methods

### Collection, identification and extraction of *Inonotus obliquus*

The detailed procedures for the collection, identification and extraction of *I*. *obliquus* has been previously described [5]. Briefly, this mushroom was collected from the Forests for the World recreation area in Prince George, British Columbia, Canada. This is public land to which no permission is required prior to accessing. These studies did not involve endangered species or protected species. Four sequential extracts were prepared using 80% ethanol (E1), 50% methanol (E2), water (E3) and 5% sodium hydroxide (E4). These extracts were further lyophilized, reconstituted in water, filter sterilized and assessed on RAW 264.7 mouse macrophage cells. All the reagents were purchased from either Sigma Aldrich Canada (Oakville, Ontario) or Thermo Fisher Scientific (Waltham, MA) unless otherwise specified.

### *In vitro* anti-inflammation assay

**Fig 1:**
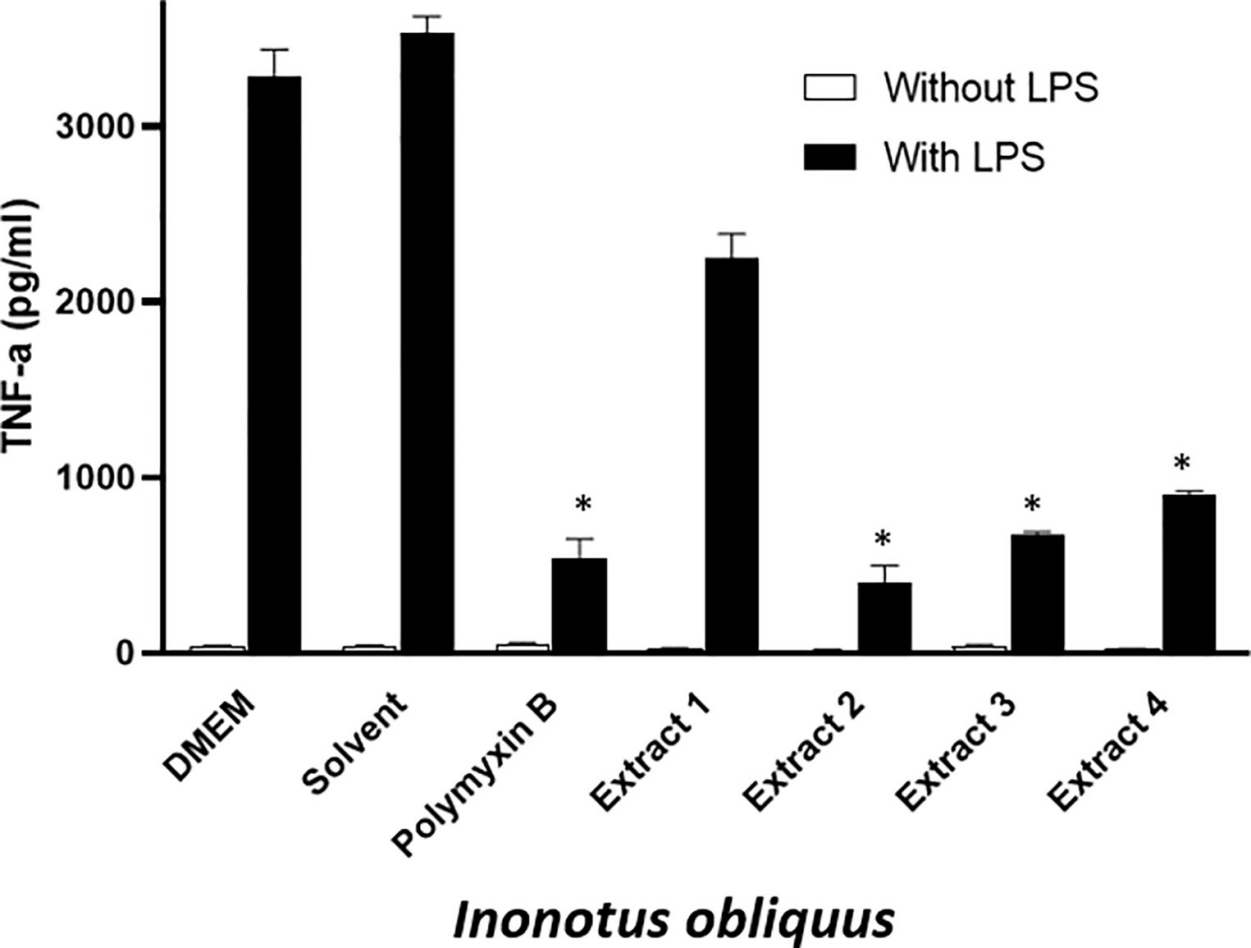
Assessing anti-inflammatory potential of *I*. *obliquus* in LPS-induced TNF-α production in RAW 264.7 macrophage cells. E1 = 80% ethanol extract, E2 = 50% methanol extract, E3 = Water extract, E4 = 5% NaOH extract. Fungal conc. 0.25 μg/μL, PMB (100 units) was used as a positive control; DMEM and solvents were used as negative controls. Error bars are standard deviations. Value are means derived from triplicates (*, P < 0.05).

Evaluation of the anti-inflammatory properties for the identified mushroom extracts was assessed using the standard protocol of inhibition of LPS-induced TNF-α production in RAW264.7 mouse macrophage cells as outlined in Fig 1. The maintenance and plating of RAW264.7 cells was previously described by Smith et al., 2017. Briefly, RAW 264.7 cells were plated in 96-wells plate (1 x 10^5^ cells/well in 200 μL of serum-free DMEM) followed by overnight incubation. On the following day, cells were washed and treated with 250 ng/ml of LPS and: (i) mushroom extracts (conc. 0.25 μg/μL), (ii) Polymyxin B (PMB; 100 units) for use as the positive control, or (iii) DMEM and water (used for re-suspension of lyophilized samples) for use as negative controls. After 6 hours of incubation, 100 μL supernatants were collected, centrifuged (4°C, 200 x g, 5 min using Allegra X-15R, benchtop centrifuge; Beckman Coulter, CA, USA) and stored at -80°C freezer until ELISA was performed for the quantification of TNF-α.

To assess histamine-induced TNF-α production and provide linkages to the future *in vivo* experiments, the following procedure was utilized. LPS was replaced by histamine to assess the effect of histamine in inducing TNF-α in RAW 264.7 macrophage cells. On the treatment day, cells were treated with histamine (1 μM) and: i) *I*. *obliquus* (E2: 1μg/mL), ii) PMB for use as the positive control, or iii) DMEM and/or water for use as negative controls. The time of treatment, the collection of supernatant and the quantification protocol (i.e. ELISA) was as above.

### Animal care & surgery

The procedures described below were an extension of protocols developed previously [24,25]. All procedures were approved by the Institutional Animal Care and Use Committee of the University of Northern British Columbia in accordance with the Canadian Council of Animal Care (CCAC) guidelines. Male black mice C57BL6 (average weight ~22–30 g and 8–44 weeks of age, n = 41) were purchased from Jackson Laboratories, Bar Harbor, ME, USA. Mice were housed at ~ 24 ^o^C on a 12:12-h light dark cycle with free access to food and water. This age of mice has previously been shown to have no significant differences [24]. The anesthetized mouse was placed prone on a transparent acrylic platform. Body temperature was held constant through indirect use of a heat lamp. The left gluteus maximus was prepared as described [24]. Viewed through a stereomicroscope, skin overlaying the left gluteus maximus muscle was removed. The proximal edge of the muscle was freed from its origin along the spine and reflected ventrally while preserving its neurovascular supply and femoral insertion. The muscle was positioned in its approximate native shape and pinned onto a pedestal of transparent Sylgard 184 (Dow Corning, Midland, MI, USA). The superfusate temperature was maintained within a perfusion reservoir (Radnoti, Monrovia, CA, USA) such that fluid over the muscle preparation was 34°C. Flow rate was maintained at ~3 mL/min using an in-line roller clamp. The muscle preparation was continuously superfused with a bicarbonate-buffered physiological saline solution (PSS; pH 7.4) of the following composition: 137 mM NaCl, 4.7 mM KCl, 1.2 mM MgSO_4_, 2 mM CaCl_2_, and 18 mM NaHCO_3_. These chemicals were purchased from Sigma Aldrich Canada (Oakville, Ontario).

#### Intravital microscopy

The completed preparation was transferred to the stage of an intravital microscope (modified model 20T, Zeiss) equilibrated for at least 60 minutes and viewed with brightfield illumination (condenser numerical aperture 0.32). mouse temperature was maintained at ~37–38 ^o^C with radiant heat. During experiments, arterioles were observed using a Zeiss UD 40 objective (numerical aperture 0.41) coupled to a video camera (model C2400, Hamamatsu); total magnification on the video monitor (model PVM-132, Sony) was 950x. Vessel diameter was determined from the outer edges with the use of a video caliper (modified model 321, Colorado Video; Boulder, CO) with spatial resolution ≤ 2 μm. Data were acquired at 40 Hz with the use of a PowerLab system (model 8S, ADInstruments; Castle Hill, Australia) coupled to a personal computer. One arteriole was studied in each mouse. Only vessels that exhibited spontaneous vasomotor tone were utilized.

### Vasoactive reagents

Chemicals and reagents were purchased from Sigma Aldrich Canada (Oakville, Ontario) or Fischer Scientific (Ottawa, ON). Final working concentrations are given after diluting stock solutions at least 10-fold in fresh PSS: Acetyl choline (ACh; 10^−9^ to 10^−5^ M; endothelium-dependent vasodilator), phenylephrine (PE; 10^−9^ to 10^−5^ M; α_1_-adrenoreceptor dependent smooth muscle contractor), histamine (10^−9^ to 10^−4^ M; NO-dependent permeability factor), C48/80 (10^−8^ to 10^−3^ M; mast cell degranulator), sodium nitroprusside (SNP, 10^−5^ M; NO donor), and pyrilamine maleate salt (10^−6^ M; H_1_ receptor antagonist).

### Microvascular dose response assessment

Experimental conditions were performed while observing second-order arterioles that were located away from the incised edge on the anterior half of the gluteus maximus preparation. During the equilibration time, signs of inflammatory reaction to histamine or C48/80, such as excessive rolling leukocytes and prolonged arteriolar dilation, were monitored. Resting diameter was measured following the equilibration period.

The effect of changing concentration of inflammatory mediators was determined by cumulative addition to the superfusion solution. At each concentration, arteriolar diameter was allowed to stabilize for at least 2 minutes and then recorded before the next increment. After recording the response at the highest concentration, superfusion with control PSS was restored and resting diameter was allowed to recover (20–30 min). The next inflammatory mediator was tested in a similar manner. Order bias was eliminated by varying the sequence of mediators in experiments; however, ACh and PE responses were always recorded prior to further testing to confirm vessel health. At the conclusion of each experiment, maximal dilation was induced and recorded through superfusion of SNP.

### Conducted vasodilation assessment

Micropipettes with internal tip diameters of 1–2 μm were pulled from borosilicate glass capillary tubes (0.69 mm ID; Harvard Apparatus) using a Sutter Instruments model P-30 micropipette puller, and backfilled with 1.0 M ACh. A filled micropipette was fitted with a rubber gasket and affixed to a stylus assembly which housed the terminal airline leading from a picoliter pressure ejection console (Warner Instruments model PLI-100A). For the purpose of the present work, the terms *upstream* and *downstream* refer to the flow direction of both PSS and blood within the test arteriole. The stylus position was adjusted using a remote-controlled motorized micromanipulator (Siskiyou model MX7600L) such that the pipette tip was positioned directly adjacent and ~5 μm above the downstream end of a 500 μm segment of a healthy second-order arteriole.

During preliminary work, it was determined that application of ~1.0 psi for 500 ms (applied through approximately 1 m of 1.5 mm ID rubber tubing) was sufficient to overcome the compression of air within the supply lines while avoiding excessive spread of ACh. An ACh pulse was applied to the test vessel accordingly. When passive ACh leakage from the micropipette was evident through flow-visualisation prior to an experiment, a hold pressure of ~0.2–0.5 psi was applied through a junction in the pressure ejection line and the ejection pressure was increased correspondingly.

To evaluate conducted vasodilation under control conditions, the vasomotor response to ACh were evaluated locally and at a remote site located ~500 μm upstream from the site of stimulus. Test vessels were allowed to return to their original resting diameter (2–3 min) before another ACh stimulus was delivered. After measurements were obtained under control conditions, test chemicals were added to the superfusate. Vessel response to ACh ejection was recorded at 10 minute intervals for 30 minutes during superfusion of a given test compound. Vessel responses were similarly recorded during washout of the test chemical using fresh PSS. Superfusion chemicals never diluted the vehicle PSS concentration below 80%, and minor variations in superfusate salt concentration during experimentation had no effect on vessel diameter or reactivity.

### Data analysis

Data are presented as mean ± standard error. Values for *n* refer to the number of arterioles studied. Each vessel response was calculated as

$$Percent\;diameter\;change=\frac{response\;diameter-resting\;diameter}{resting\;diameter}\times 100\%$$

A one-way repeated measures analysis of variance with Tukey post hoc comparison was used to compare mean values among treatment groups (Minitab 17 Statistical Software; State College, PA, USA). Differences among groups were considered statistically significant with *P* < 0.05.

## Results

### Assessing the effects of crude extracts from *I*. *obliquus* on *in vitro* anti-inflammation assay

To assess for anti-inflammatory activity *in vitro*, RAW264.7 cells were treated with 250ng/mL of LPS plus 0.25 μg/μL *I*. *obliquus* extracts. Polymyxin B (PMB: active ingredient of reputed anti-inflammatory ointment-Polysporin) was used as a positive control for the estimation of a relative anti-inflammatory potential of wild fungi. Fig 1 showed that PMB significantly inhibited TNF-α induced by LPS. Moreover, consistent with our previous findings [5], all four extracts of *I*. *obliquus* demonstrated potent anti-inflammatory activity, even at 0.25μg/μL.

**Fig 2:**
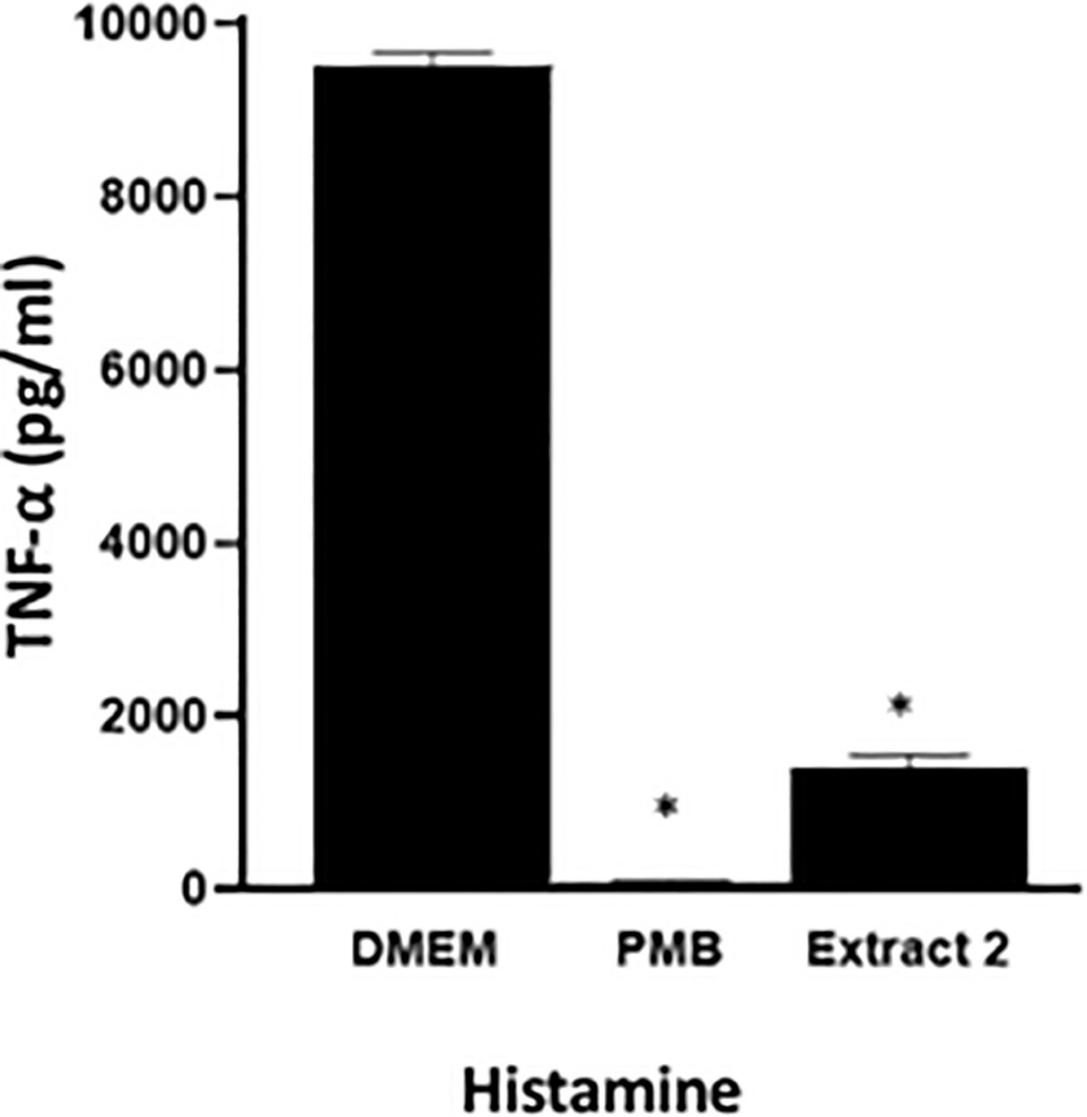
Inhibition of histamine-induced TNF-α production. In RAW 264.7 macrophage cells the mushroom extract *I*. *obliquus* (E2) was used. PMB (100 units) was used as a positive control and DMEM was used as negative control. The histamine concentration used is the dose for inducing maximum TNF-α- was 10^−6^ M. Error bars represent standard deviation (*, P < 0.05).

Histamine has been used as an induce inflammation in *in vivo* models of microcirculation [12, 26]. To simulate a similar condition in RAW 264.7 macrophage cells, LPS was replaced by histamine. One μM of histamine was selected as it triggered the maximum concentration of TNF-α production. The *I*. *obliquus* E2 extract (1μg/μL) was treated in the presence of histamine to observe its potential inhibitory effect in the presence of an inflammatory mediator. Fig 2 shows that E2 extract of *I*. *obliquus* significantly inhibited histamine-induced TNF-α by > 90%, indicating its strong potential in treating inflammation. For the remainder of the outlined results *I*. *obliquus* refers to the E2 extract.

### Assessment of microvascular health

Before proceeding to documenting the effects of *I*. *obliquus* on the microcirculation in mice, a number of studies to optimize the experimental conditions would first need to be established. These include evaluating the vascular tone of arterioles to ACh and PE, and obtaining the histamine dose-response curve. For determining the baseline and maximum diameters, second-order arterioles (2A) were studied in male C57B76 mice. Minimal variation was observed in the resting diameter of 2A arterioles of the mice. The average resting diameter was calculated as 19 ± 2 μm (n = 10). The maximum dilation of the test vessel in response to ACh (10^−4^ M) equals 38 ± 2 μm (n = 5), and that of SNP (10^−2^ M) equals 37 ± 3 μm (n = 10). There is approximately 151% change between the resting and the maximum diameter. Table 1 displays the graphical representation of minimum (resting) and maximum (ACh and SNP) diameters changes. The data are presented as mean ± SEM.

**Table 1:** Baseline (resting) and maximum diameters (ACh and SNP) in 2A arteriole.

Parameters	Vessel Diameter (μm)	n
Baseline (Resting)	19 ± 2	10
Acetylcholine (ACh) 10^−4^ M	38 ± 2	5
Sodium Nitroprusside (SNP) 10^−2^ M	37 ± 3	10

**Fig 3:**
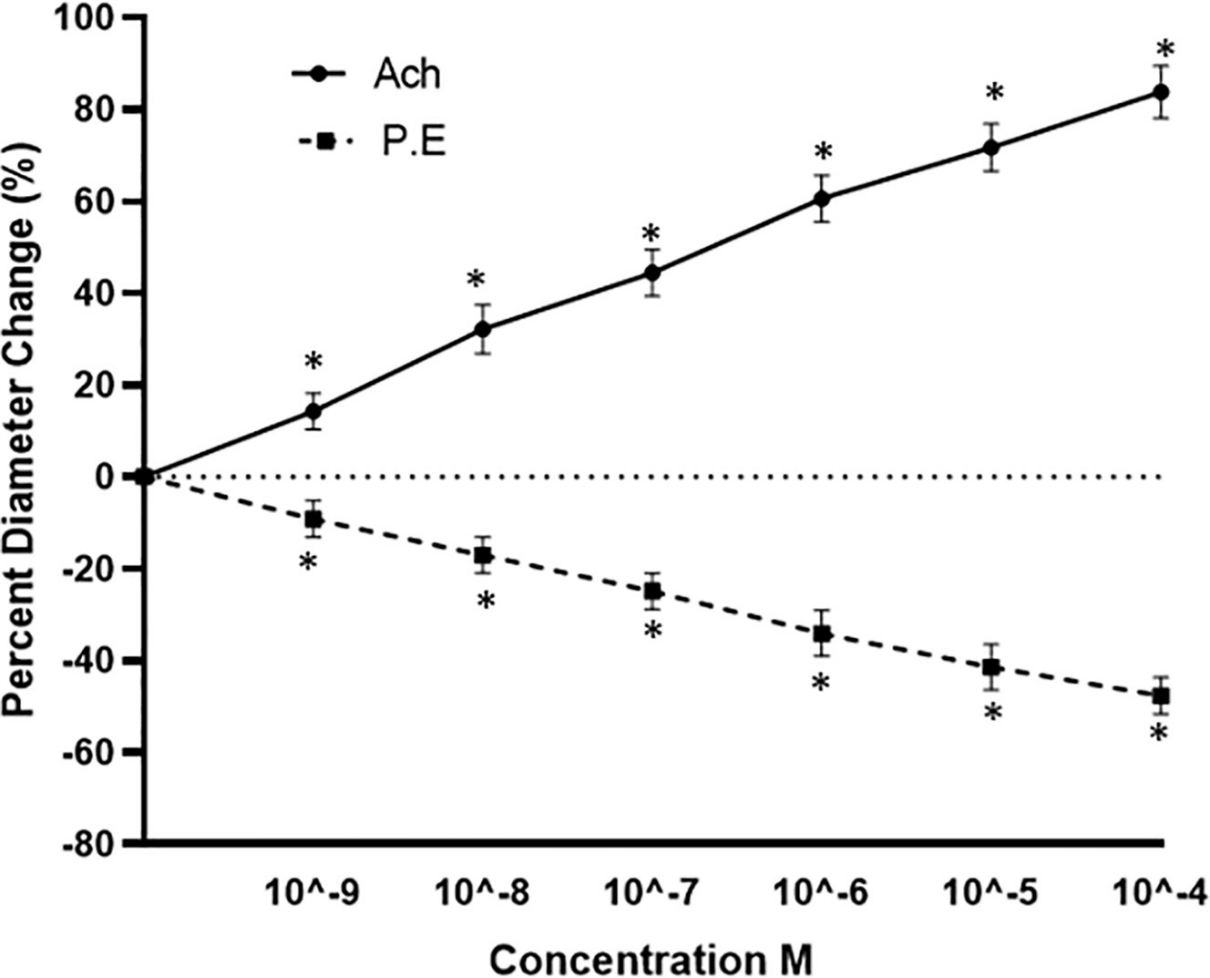
Dose response curve of Acetylcholine (ACh) and Phenylephrine (PE) on second-order arterioles of gluteus maximus of male C57BL/6 mice *(n =* 10). Values represent mean (± SEM) vessel diameter change calculated based on peak dilation or constriction as a percentage of resting diameter.

**Fig 4:**
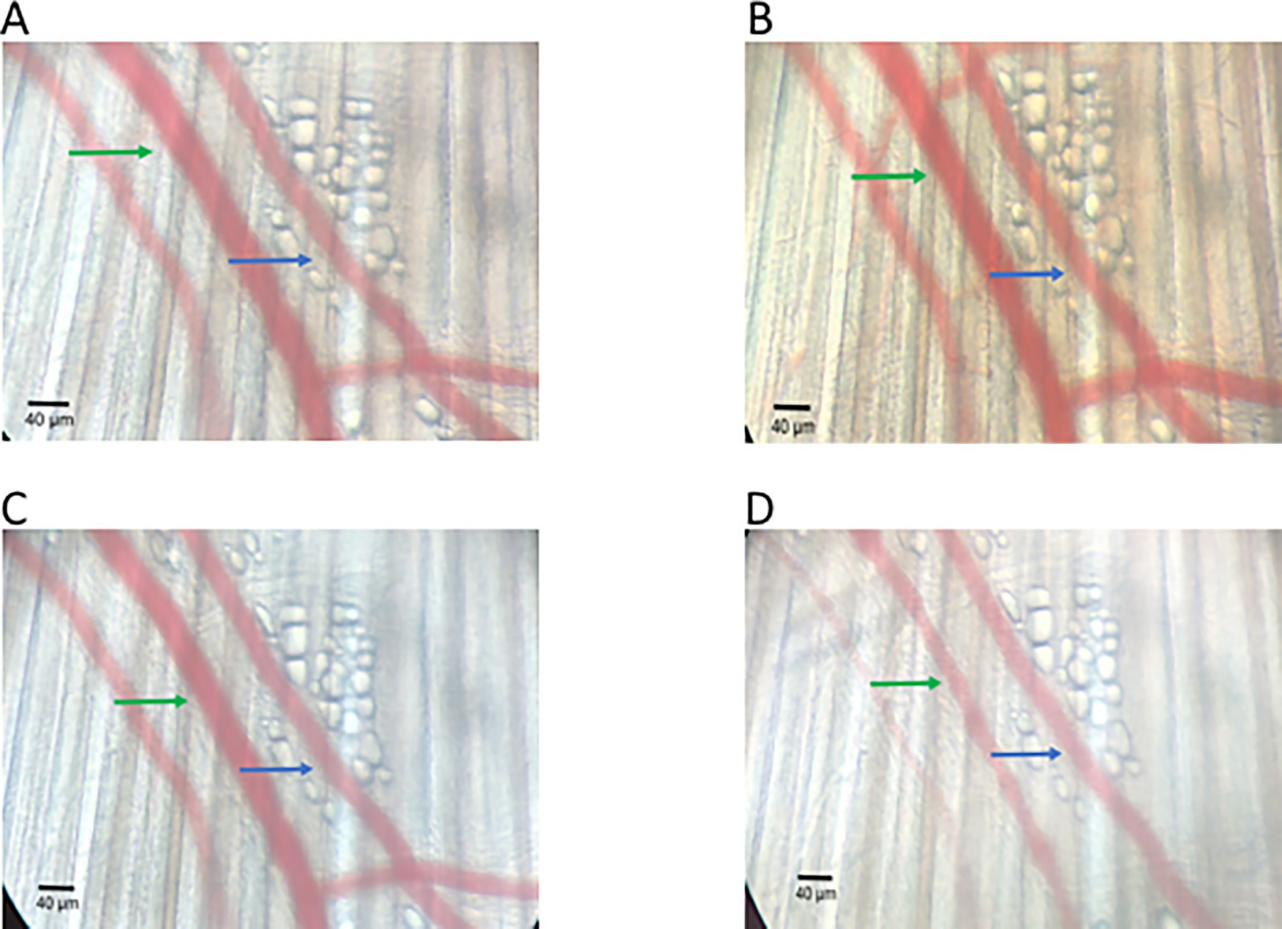
Representative changes of arteriolar diameter following exposure of ACh or PE. (A) baseline, (B) ACh 10-5M, (C) baseline, (D) PE 10-5M. Green arrow (arteriole), Blue arrow (Venule) and scale bar (μm).

For evaluation of the vascular tone, gluteus maximus muscle was subjected to different doses of ACh and PE (10^−9^ to 10^−4^ M). Fig 3 demonstrated the successful induction of vasodilation and vasoconstriction in the arterioles with the help of ACh and PE respectively in a dose-dependent manner. The arteriole increased steadily by raising the dose of ACh. The percent change of diameter from resting diameter decreased gradually by increasing the dose of PE in the superfused solution. These results indicate that the endothelium and smooth muscles in the arterioles were intact and reactive to the added molecules and the doses of vasoactive chemicals were sufficient to elicit a response. Representative images highlight this is Fig 4A (baseline vessel) and Fig 4B (Ach 10-5M) to highlight a robust vasodilation. Additionally, Fig 4C (baseline vessel) and Fig 4D (PE 10-5M) demonstrate robust vasoconstriction.

**Fig 5:**
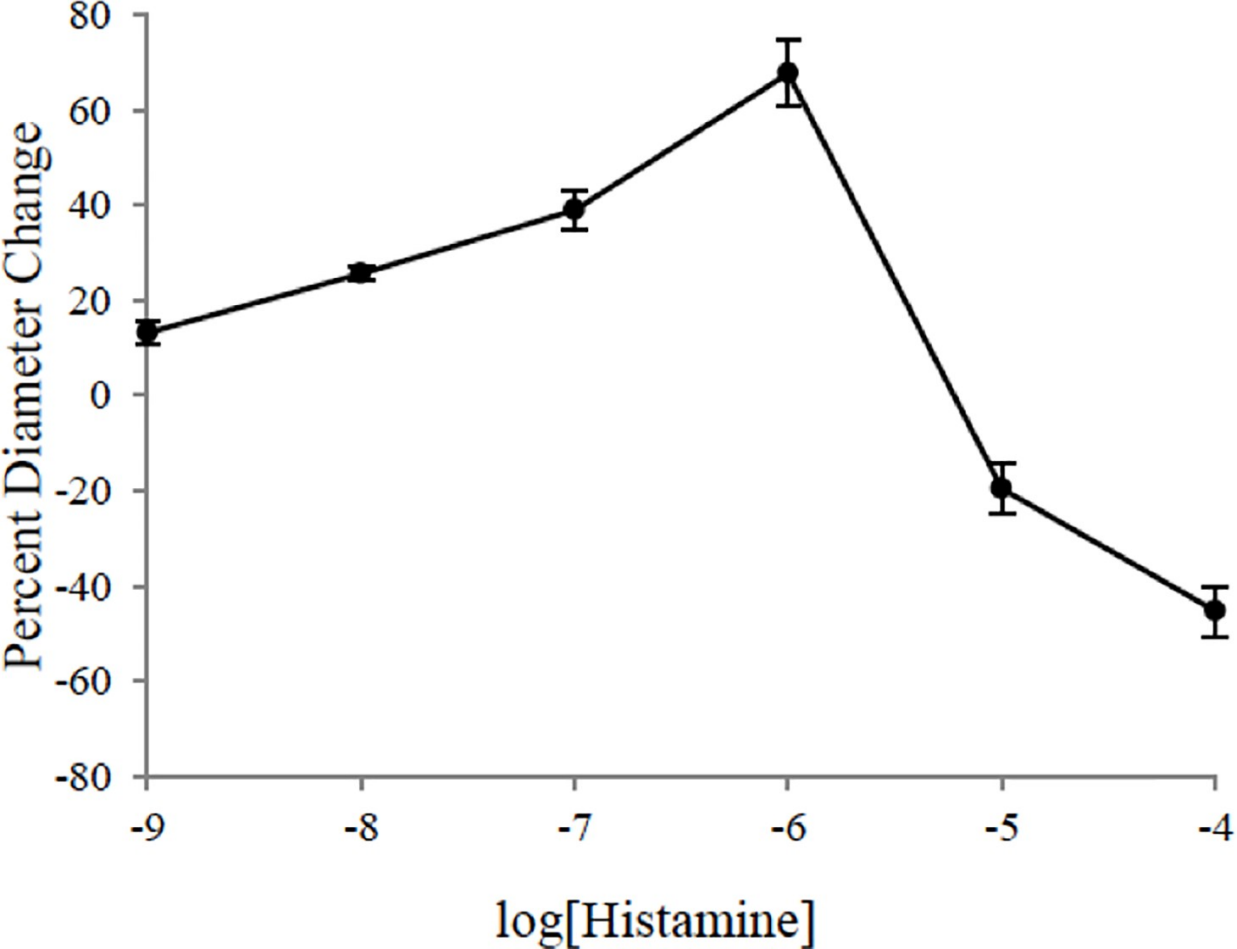
Dose response curve of histamine on second-order arterioles of gluteus maximus of male C57BL/6 mice *(n = * 5). Values represent mean (± SEM) vessel diameter change calculated based on peak dilation or constriction as a percentage of resting diameter.

Histamine was used to induce local inflammation in the 2A arteriole in a dose-dependent manner. Fig 5 illustrates the effect of histamine (10^−9^ to 10^−4^ M) on the arteriolar reactivity. The dose-response curve demonstrates a bi-phasic reaction in which the vessels vasodilated when subjected to 10^−9^ to 10^−6^ M histamine, and vasoconstricted when subjected to 10^−5^ to 10^−4^ M histamine. In order to have an observable vascular tone while still exerting an inflammatory effect, 10^−5^ M for conducted vasodilation experiments.

**Fig 6:**
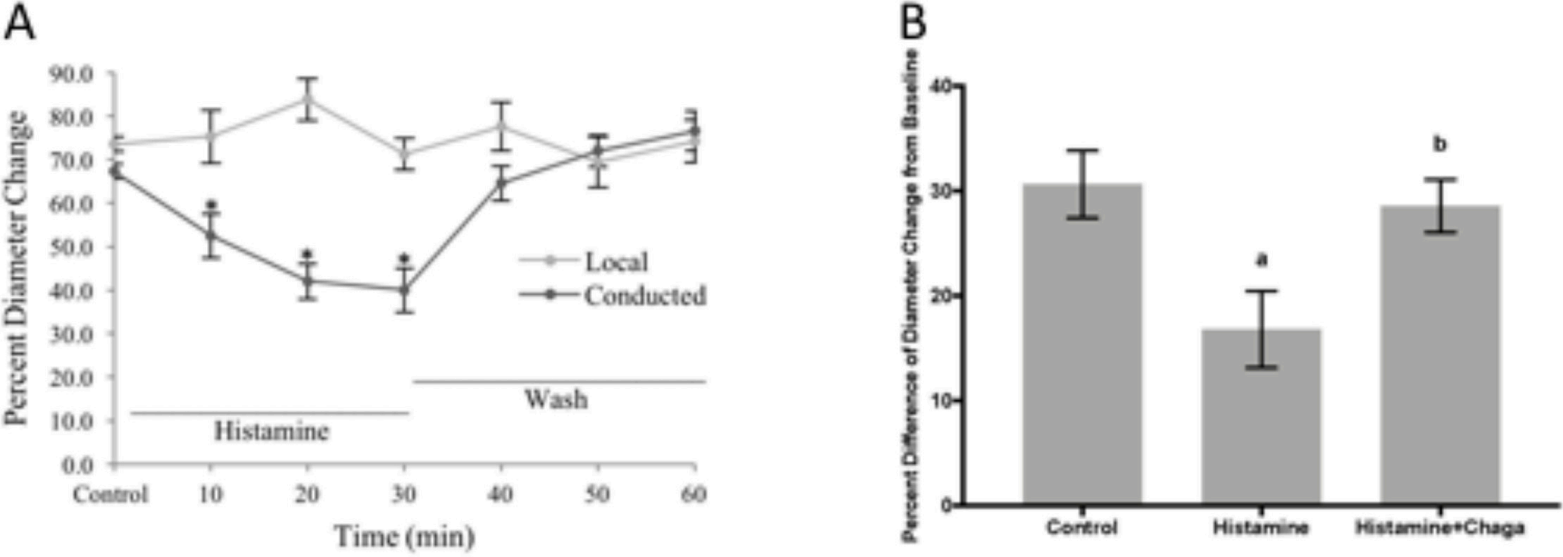
Conducted vasodilation in arterioles of C57BL6 mice (n = 5). **(A)Responses to ACh (1psi, 500 ms) were recorded at the remote site upstream (with respect to blood flow) along the arteriole through a distance of 500 μm (conducted response).** At each site, “diameter change” was calculated as peak response diameter–resting diameter. (B) *I*. *obliquus (*Chaga*)* reverses the reduction of the conducted response by histamine. There is significant difference between the control conducted response and histamine (a; p <0.05) and reversal of histamine vs. histamine + *I*. *obliquus* (Chaga) (b; *P* <0.07).

To evaluate *I*. *obliquus* extract on Conducted Vasodilation (CVD), various doses of E2 extract were tested. The dose selected for the CVD experiment was 12.5 μg/mL because at this concentration the vessels were minimally active (S1 Fig). As previously shown [26] histamine significantly impairs conducted vasodilation. Our results in this study are identical to our previous work (Fig 6A) with no change at the local site indicative of the reduction of conducted vasodilation as a result of loss of microvascular junctional integrity and not a reduction of the local stimulus to ACh [27]. The loss of conducted vasodilation is reversed upon washout of histamine and restoration of the conducted vasodilation response. There is no significant impact of either the local response to ACh or conducted with *I*. *obliquus* (E2) alone. However, as Fig 6B demonstrates, the CVD of *I*. *obliquus* (E2) when added with histamine to explore its role during an anti-inflammatory event was able to restore the decreased conducted vasodilation response to 28.6 ± 2.5% (*I*. *obliquus* (E2) + histamine) from 16.8 ± 3.7% (histamine alone; S2 Fig).

**Fig 7:**
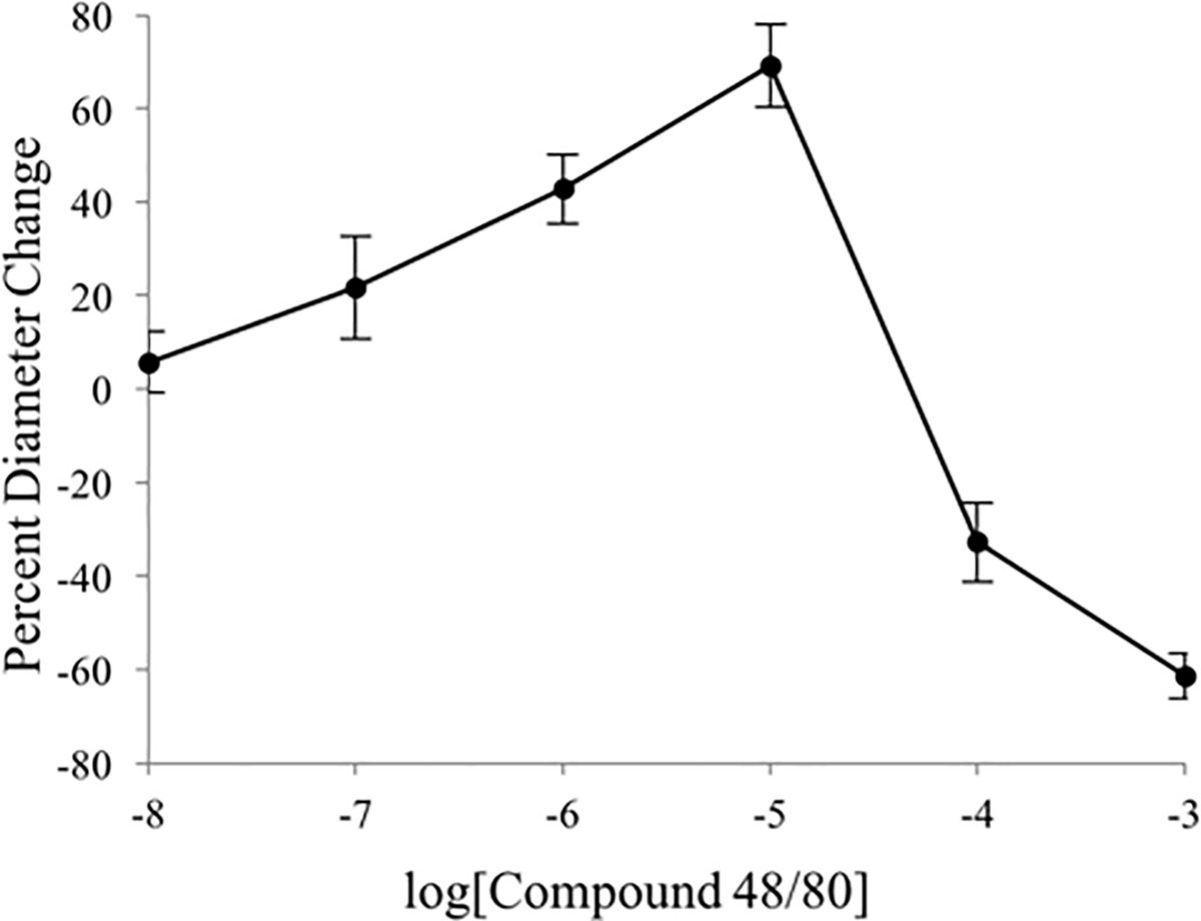
Vasoactivity of compound 48/80 on second-order arterioles of gluteus maximus of male C57BL/6 mice. C48/80 exhibits a biphasic dose-response pattern. Values represent mean (± SEM) vessel diameter change calculated based on peak dilation or constriction as a percentage of resting diameter **(*n* = 5)**.

To assess the role that mast cells play in histaime-induced reduction of CVD we used the mast cell degranulator C48/80. Data showed that C48/80 had a similar biphasic dose-resoponse pattern to histamine. Test arterioles dilated at superfusate C48/80 concentrations of between 10^−9^ and 10^−6^ M, and constricted at 10^−5^ and 10^−4^ M. Peak dilation (69% change from resting baseline) occurred at 10^−5^ M. There was a mild constriction below resting diameter at 10^−4^ M and a strong constriction leading to complete vessel closure at 10^−3^ M (Fig 7).

Interestingly, the dose-response relationship of C48/80 has a high degree of similarity to that of histamine, but takes place at a 10-fold greater concentration. There is a more pronounced constriction at the highest doses of C48/80 (-33 ± 8% and -61 ± 5%) than for the highest doses of histamine (-20 ± 5% and -45 ± 5%). C48/80 in solution was manageable at low concentrations, but as the concentration increased to the mM range, a surface film and excessive bubbling formed within the superfusate, potentially compromising the uptake of C48/80 into the preparation.

**Fig 8:**
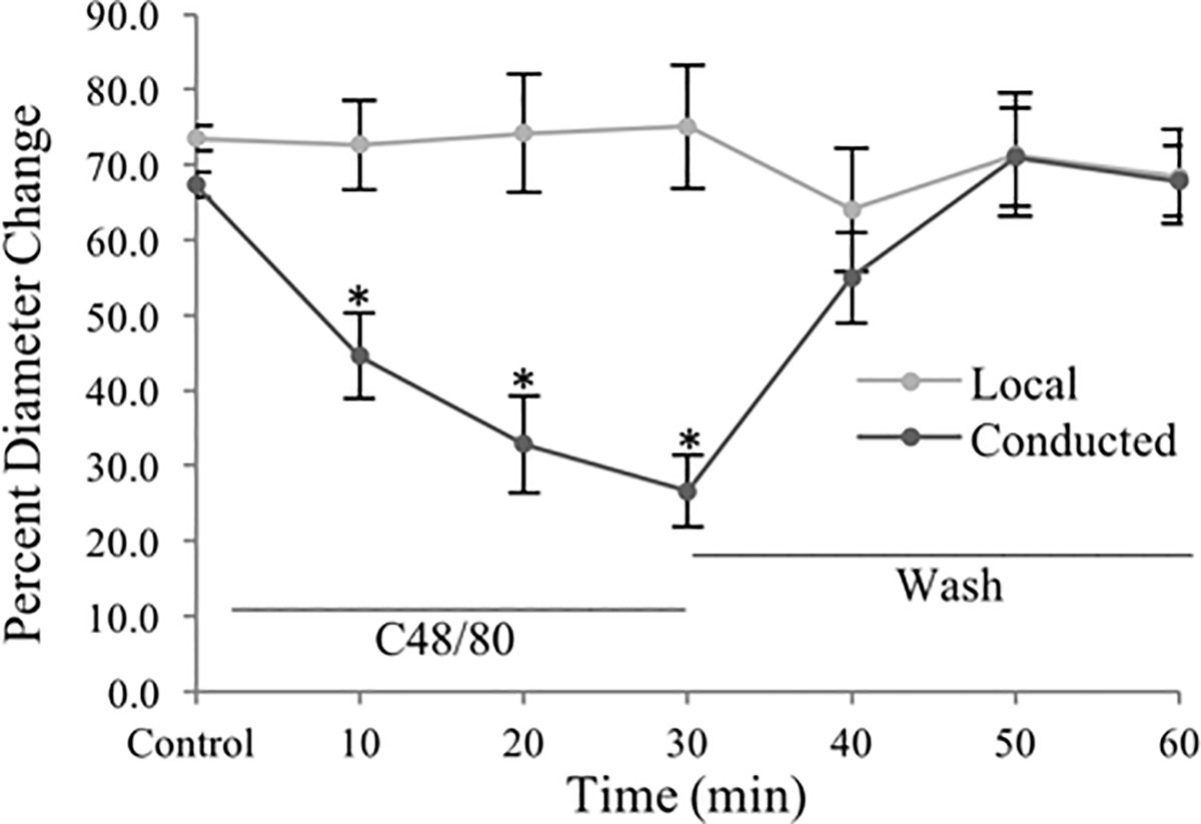
Local and conducted vasodilation of second-order arterioles of male C57BL/6 mouse gluteus maximus. In response to 1 M Ach in the presence of 10^−6^ M compound 48/80 the conducted vasodilation response is reduced and restored upon washout of the compound (*n* = 5). Values represent mean (± SEM) vessel diameter change calculated based on peak dilation or constriction as a percentage of resting diameter. *P < 0.05 vs. local dilation.

Conducted vasodilation was diminished upon exposure to 10^−6^ M C48/80 (Fig 8) similarly to histamine treatment. The effect peaked at an upstream vessel diameter change 41% below control. Washout of C48/80 reestablished conducted vasodilation to control levels within 10–20 min. No significant difference in local dilation appeared during experimentation.

**Fig 9:**
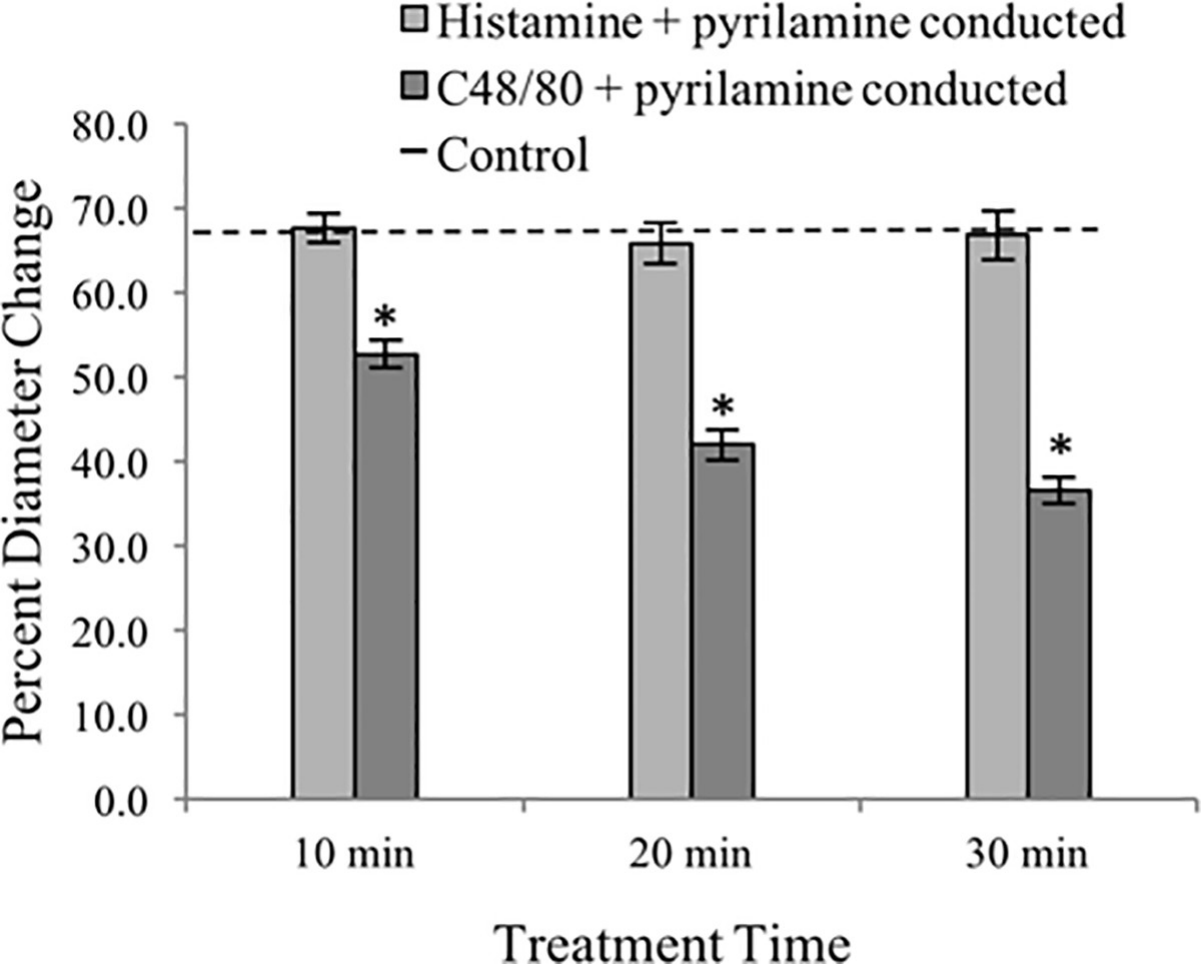
Effect of histamine and compound 48/80 on the conduction of vasodilation in the presence of pyrilamine. Pyrilamine abolished the effect of histamine on reducing conducted vasodilation but combination of C48/80 and pyrilamine, a significant reduction in conducted vasodilation was still observed. Values represent mean (± SEM) vessel diameter change calculated based on peak dilation or constriction as a percentage of resting diameter. Control value represents conducted dilation of vessels prior to treatment (*n* = 4 for each treatment). *P < 0.05 vs. control and histamine + pyrilamine treatment.

To assess the role of the histamine H1 receptor activation plays in mediating the impact of reducing the conducted vasodilation by either histamine or C48/80 we measured the effect of in the presence of the H1 receptor antagonist pyrilamine (10^−6^ M). This revealed an interesting distinction (Fig 9). Pyrilamine abolished the effect of histamine; conducted vasodilation remained within 2% of the control value for the duration of the treatment. However, during treatment with a combination of C48/80 and pyrilamine, a significant reduction in conducted vasodilation was observed. The effect reduced dilation to a minimum of only 36.6% at 30 minutes. Though not significant at α = 0.05, it is worth noting the reduction in conducted vasodilation from C48/80 alone (Fig 7) was ~10% greater at each interval than when combined with pyrilamine (Fig 9). There was no significant difference in local dilation during any of the pyrilamine treatments (not displayed).

## Discussion

Previous work with *Inonotus obliquus* has demonstrated anti-inflammatory activity [5,7,8,9,10] and in this study, we showed that all four extracts of *I*. *obliquus* collected were able to significantly inhibit LPS-induced TNF-α production in RAW 264.7 mouse macrophage cells (Fig 1). These findings are consistent with previously published research where extracts and active constituents isolated from *I*. *obliquus* have inhibited LPS-induced macrophages by decreasing the hyperactive release of inflammatory cytokines such as TNF-α, Nitric oxide, COX-2, among others.

For the use of histamine-induced TNF-α *in vitro* experiments, the *I*. *obliquus* extract successfully inhibited the induced TNF-α production and therefore has shown similar results as were shown in experiments with LPS-induced TNF-α production (Fig 2). This experiment was used to simulate *in vivo* physiological conditions (i.e. histamine-induced inflammatory event as opposed to LPS-induced inflammatory response). However, to our knowledge, this study is the first to induce the production of TNF-α using histamine in mouse macrophages RAW 264.7 cells. There is one similar study in human liver macrophages in which histamine was used to induce exocytosis and to stimulate the release of pro-inflammatory cytokines (IL-6). Interestingly, in their study, 10^−6^ M histamine stimulated the maximum release of the cytokine, which is consistent with the current study [27]. Additionally, *I*. *obliquus* was successful in treating carrageenan-induced edema in rats and colitis-associated inflammation in an *in vivo* mouse model [6–9].

The *in vitro* assessment *I*. *obliquus* of clearly indicates that there are clear anti-inflammatory properties and that the methanol extract (E2) has the most potent anti-inflammatory activity in inhibiting histamine-induced TNF-α production in RAW 264.7 cells.

Expanding from the *in vitro* assessment, we assessed the potential positive impact of *I*. *obliquus* on second-order mouse gluteus maximus arterioles. The anterior section of the gluteus maximus preparation was favoured when selecting test vessels. This was to control for any potential effect resulting from the destruction of the inferior gluteal nerve during surgery. Baseline data of microvascular health prior to both inflammatory assessment indicated the overall health of the microcirculation of these arterioles. The vasomotor response to ACh and PE (Fig 3) indicated that test vessel health was maintained well throughout preparation of the gluteus maximus. Response trends are similar to those found previously, and the comparable response to both SNP and the highest dose of ACh is indicative of intact endothelium-dependent smooth muscle relaxation [24].

In addition to the ACh and PE responses, histamine produces a trend similar to that described previously [12]. The biphasic response observed in the cremaster preparation was reproduced here in the gluteus maximus, with vasodilation increasing in intensity up to 10^−6^ M, followed by vasoconstriction at 10^−5^ M and higher (Fig 5). It was shown previously through genetic and pharmacological ablation of eNOS that histamine induced dilation is purely a result of NO release [12]. Furthermore, it was shown that both dilation and constriction are mediated via H_1_ receptors, with vasodilation dependent on NO production by endothelium and vasoconstriction promoted through the activation of L-type calcium channels in smooth muscle. The simultaneous activation of both cell layers provides modulation of the vasomotor response that is dependent on local histamine concentration. That is, the normal response to histamine is to promote blood flow into the inflamed muscle, but with a strong release of histamine, arteriolar constriction reduces capillary hydrostatic pressure and muscle blood flow. This protective mechanism would become pronounced as shear stress within the blood vessel weakens the coupling between endothelial cells, halting endothelial cell-mediated smooth muscle relaxation and reducing fluid extravasation during injury and inflammation [12].

The integral component to anti-inflammatory action by *I*. *obliquus* would be determine whether histamine induced reduction of conduction vasodilation would be altered by *I*. *obliquus* treatment. It is clear that *I*. *obliquus* reverses the negative impact histamine-induced reduction of conducted vasodilation (Fig 6A) and restores to normal conducted vasodilation levels in the presence of histamine (Fig 6B). This is a clear indication that the anti-inflammatory properties of *I*. *obliquus* observed in vitro are similar in effect *in vivo*.

This model involved live imaging of the inflammatory event and observation of the improvement in the inflammatory event in real time. The *in vivo* results indicated that, as the cellular communication was blocked up-stream by histamine-induced inflammatory events, the mushroom extract was able to normalize the abated communication when tested with histamine. To our knowledge, this study is the first of its kind to study mushroom extracts for their anti-inflammatory activity in mice micro-circulation, which provides the basic framework to abridge the concept of live vessels for natural products assessment leading to drug discovery.

The novel characterization of C48/80 performed in the present study revealed an interesting similarity to the vasoactivity of histamine. However, the inflection from dilation to constriction takes place at a 10-fold greater concentration (10^−4^ M; Fig 7). It would be reasonable to question whether the vasoactivity shown here is simply a result of histamine released via mast cell degranulation. However, it is more likely that there exists at least some degree of cooperation between the products of mast cell degranulation. Besides releasing histamine to increase vascular permeability, mast cells release proteinases, heparin, serotonin, prostaglandins, and chemotactic factors [28]. Characterization of C48/80 showed similar inhibition of conducted vasodilation, but interestingly demonstrated a more pronounced effect (Fig 8). This lends support to the notion that histamine is just one of many compounds that contribute to inflammatory processes within the vasculature.

In order to address the possibility that histamine released from mast cells during equilibration of C48/80 was responsible for the similarity in dose-response patterns, a combination of C48/80 and the H_1_ receptor antagonist pyrilamine was tested for its effect on conducted vasodilation (Fig 9). Since the reduction of conducted vasodilation was reproduced in the presence of pyrilamine, it is suggested here that H_1_ receptor agonists only account for part of the reduction of CVD observed with C48/80. This supports previous work utilizing a rabbit aorta model [29].

The present study revealed that the biphasic histamine dose-response curve demonstrated here and in previous work [12] is reproduced by the mast cell degranulator C48/80. Further similarity was shown in both impairment of conducted vasodilation and recovery upon washout. A prominent difference between the two inflammatory mediators was uncovered when the H_1_ receptor antagonist pyrilamine was added to the superfusate. Pyrilamine abolished the effect of histamine, but did not significantly reduce the effect of C48/80. This supports the notion that histamine is a major effector of junctional integrity along the vascular endothelium [26], and broadens the scope of inflammatory inhibition of conduction from histamine to a host of other mediators. Indeed, data presented here suggests histamine may only be partly responsible for the putative inhibition of conducted vasodilation.

The well characterized age-related decline in vascular function [12] offers a glimpse into the relevance of blood flow control in disease states. Inflammatory deterioration of conducted vasodilation ultimately reduces the capacity for fine control over blood flow and likely diminishes the capability for maintenance and growth of skeletal muscle. Here, adapting previous work performed in the cremaster allowed for greater inference into disease states, given that the gluteus maximus is a muscle present in both genders, and is heavily recruited in locomotion. Beyond age-related deterioration, inflammation and mast cell activity have shown some degree of correlation with inflammatory arthritis, obesity, diabetes, and cardiovascular diseases such as atherosclerosis [17,18,19,27] However, the reduced skeletal muscle performance alluded to here may contribute to the sedentary lifestyles which have been shown to promote chronic inflammation [30]. Undoubtedly, further research aimed at translating the intravital vascular responses demonstrated here to various disease states is warranted.

The current study builds upon the findings from the previous studies and explores the role of wild mushrooms demonstrating potent anti-inflammatory effects. As microcirculation acts as an integral component of the inflammatory response, it is critical for any therapeutic target to maximize the targeted efficacy of any anti-inflammatory drug at the microvasculature level. This study provides a framework for further exploring mushroom extracts in both an *in vitro* and *in vivo* model. Future studies could explore the specific mechanism of action of these extracts in perfecting the diminished conducted response. Following the model study by Looft-Wilson et al., 2004 [25] which was one of the first studies to assess the role and pattern of connexin expression in the microvasculature, which comprise gap junctions, and integral to the conducted vasodilation response. Future studies could also consider the role of the special gap junction protein, Connexin, and its three isoforms (i.e. Cx37, Cx40, and Cx43) is diminished (i.e. inflammation) and re-established (i.e. anti-inflammation) conducted response as a result of *I*. *obliquus*. More extensive studies could also involve the use of inflammation-induced mouse models (e.g. colorectal cancer mouse model) to study the effect of potent extracts in treating inflammation-associated tumors which will be helpful in the prevention and treatment of chronic inflammatory states and the tumors associated with these hyperactive immune conditions. This will be helpful in elucidating new mechanisms of combating inflammation at the cellular level.

In summary, all four extracts of *I*. *obliquus* possess anti-inflammatory effects in reducing LPS-induced TNF-α in RAW 264.7 cells. The methanolic extract (E2) demonstrated the most potent activity and was also able to significantly inhibit histamine-induced TNF-α production in RAW 264.7 cells. This extract was then tested in mouse microcirculation model in which it was able to reverse the decrease in conducted vasodilation response during inflammation. Further, there may be a role for mast cells as a target in future studies in highlighting therapeutic potential targets for medicinal mushrooms.

## Supporting information

S1 Fig Vasoactivity of Chaga (*I*. *obliquus)* on second-order arterioles of gluteus maximus of male C57BL/6 mice. Values represent mean (± SEM) vessel diameter change calculated as constriction as a percentage of resting diameter (n = 3).
(TIFF)

S2 Fig Representative traces. Illustrating local vasodilation to ACh (left trace) but histamine-inhibited conducted vasodilation (middle traces) and restoration of conducted vasodilation response in the presence of histamine and *I*. *obliquus*. Arrows in each trace indicate delivery of ACh stimulus at local site.
(TIFF)
