# Supplementary figures and images for: Inonotus obliquus attenuates histamine-induced microvascular inflammation
Source: PLoS One. 2019 Aug 22;14(8):e0220776. doi: 10.1371/journal.pone.0220776 (PMC6706056; doi:10.1371/journal.pone.0220776)

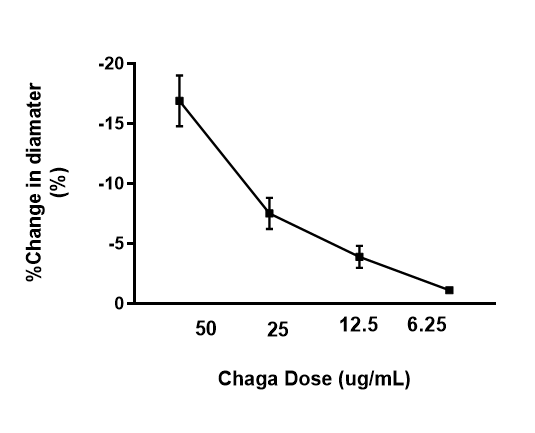

Supplement: S1 Fig — Values represent mean (± SEM) vessel diameter change calculated as constriction as a percentage of resting diameter (n = 3). (TIFF) [file pone.0220776.s001.tiff]

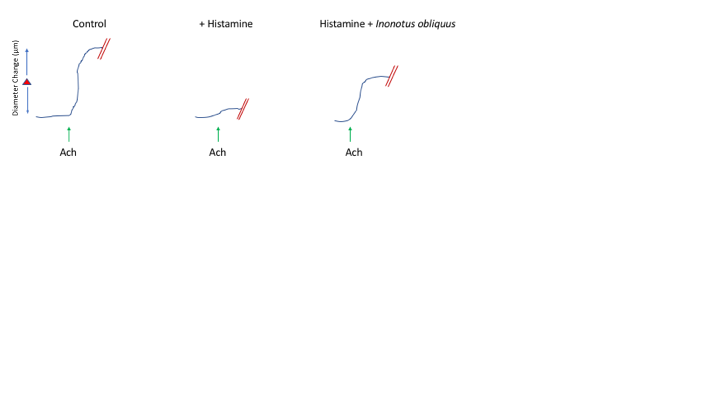

Supplement: S2 Fig — Illustrating local vasodilation to ACh (left trace) but histamine-inhibited conducted vasodilation (middle traces) and restoration of conducted vasodilation response in the presence of histamine and I. obliquus. Arrows in each trace indicate delivery of ACh stimulus at local site. (TIFF) [file pone.0220776.s002.tiff]
